# A Retrospective Analysis of Risk Factors of COVID-19 Associated Mucormycosis and Mortality Predictors: A Single-Center Study

**DOI:** 10.7759/cureus.18718

**Published:** 2021-10-12

**Authors:** Kapil Zirpe, Prajakta Pote, Abhijit Deshmukh, Sushma K Gurav, Anand M Tiwari, Prasad Suryawanshi

**Affiliations:** 1 Neuro Trauma Intensive Care Unit, Ruby Hall Clinic, Pune, IND

**Keywords:** diabetes, mortality, black fungus, covid-19, mucormycosis

## Abstract

Background

Mucormycosis has been identified with increasing frequency in patients with coronavirus disease 2019 (COVID-19).

Aims

We aimed to determine the in-hospital outcome of patients with COVID-19 associated mucormycosis (CAM).

Materials and methods

This was a single-center, retrospective, observational study. We included patients diagnosed with CAM from a tertiary care hospital in Pune, India. Clinical, laboratory, and in-hospital outcomes were noted. We analyzed factors associated with in-hospital mortality.

Results

Between February 2021 and June 2021, we identified 84 patients of CAM. The mean age was 49.3 ± 12.1 years. Of the included patients, 64.3% had diabetes mellitus, and 83.3% had received steroids. Mucormycosis was diagnosed after a median of 11 days from the COVID-19 diagnosis. Orbital and central nervous system (CNS) involvement was seen in 29.8% and 23.8% of patients, respectively. During a mean hospital stay of 12.5 ± 8.5 days, 15.5% of patients died. Compared to survivors, the presence of chronic kidney disease (CKD) (p<0.0001), orbital involvement (p=0.039), use of tocilizumab (p<0.0001), and development of renal dysfunction during hospitalization (p<0.0001) were seen in a significantly higher proportion of nonsurvivors. The proportion of patients with diabetes, those receiving steroids, and mean glycosylated hemoglobin (HbA1c) levels did not differ significantly in survivors and nonsurvivors.

Conclusion

In-hospital mortality in CAM is relatively lower in our institution. CKD, orbital involvement, use of tocilizumab, and renal dysfunction during hospital stay were found to be strong predictors of mortality.

## Introduction

Over the last 19 months, the world has witnessed the devastating coronavirus disease 2019 (COVID-19) pandemic that has caused significant morbidity and mortality [1]. Though most patients with COVID-19 have mild to moderate disease, numerous risk factors, including old age and comorbidities (e.g., obesity, cancer, diabetes), predispose people to severe disease [2,3]. Management varies depending on the severity of the condition, and the best treatment for COVID-19 is still unknown. However, mortality reduction was observed with the use of steroids in hospitalized patients [4]. In the second wave of the COVID-19 pandemic, India witnessed a significant increase in the incidence of COVID-19 associated mucormycosis (CAM) and contributed to over 70% of CAM cases worldwide [5]. Globally, the incidence of mucormycosis in the general population varies from 0.005 to 1.7 per million population. However, India has an 80 times higher prevalence of mucormycosis than developed countries [6]. CAM is observed in patients with ongoing COVID-19 or during convalescence. CAM risk factors include uncontrolled diabetes, respiratory diseases including viral/bacterial infections, malignancy (e.g., hematological), and the immunocompromised state [7,8]. Mortality rates vary substantially in reported studies of CAM [7,9]. A systematic review of 101 cases reported a mortality rate of 30.7% [8]. However, studies assessing the predictors of mortality in CAM are lacking. In this study, we aimed to determine the predictors of in-hospital mortality in patients with CAM.

## Materials and methods

In this single-center, retrospective, observational study, patients diagnosed with CAM were recruited from a tertiary level intensive care unit from Pune, India. Equipped with modern facilities, our ICU provides the tertiary level of intensive care to urban and semi-urban populations. The study was conducted according to the principles of the Declaration of Helsinki and good clinical practice, and local applicable regulatory guidelines. The institutional ethics committee (biomedical and health research) of Poona Medical Research Foundation (institutional review board) approved the study. The consent was waived as this was a retrospective study.

In this study, we scrutinized our patient database between February 2021 and June 2021 to identify patients diagnosed with CAM. All these patients were referred to us from other centers with suspicion of CAM. They were clinically diagnosed and confirmed at our center. CAM was defined by the presence of mucormycosis in proven COVID-19 patients. COVID-19 diagnosis was based on the reverse transcription-polymerase chain reaction (RT-PCR) test positivity on nasal and/or pharyngeal samples. Mucormycosis diagnosis was based on presenting clinical symptoms like eye pain, headache, facial swelling combined with appropriate imaging such as computerized tomography of the paranasal sinus (CT-PNS) or magnetic resonance imaging of paranasal sinus (MRI-PNS) and demonstration of fungi by methods such as screening by potassium hydroxide (KOH) mount, fungal culture, and histopathology. We included adult patients aged 18 years and above of either gender who had a diagnosis of CAM and were treated with operative procedures and pharmacotherapy. All CAM cases had diligent blood sugar monitoring and good intra-hospital glycemic control with multidisciplinary medical care.

Data on demographic, clinical, and outcomes parameters from the patient record files were identified and noted in a structured case record proforma. Clinical data for COVID-19 related parameters, such as the presence of comorbidities, need for hospitalization, use of steroids and immunomodulators such as tocilizumab, were noted. Clinical symptoms and signs of CAM were also captured. Data of laboratory parameters such as hemoglobin, total leucocyte count, glycosylated hemoglobin (HbA1c), serum creatinine, and inflammatory markers such as C-reactive protein (CRP), D-dimer, ferritin, etc., were also captured. Renal dysfunction was diagnosed as patients with known chronic kidney disease (CKD) or patients who developed acute kidney injury (AKI) according to 2012 KDIGO guidelines of AKI [10]. Based on MRI grade, CAM cases were classified for rhino-orbital-cerebral mucormycosis (ROCM), as shown in Table 1 [11]. The primary outcome was mortality during the stay in a hospital. We assessed the mortality rate and factors that predicted mortality.

**Table 1:** Proposed staging of rhino-orbital-cerebral mucormycosis (ROCM)

Grade	Description
Grade 1	Nasal involvement
Grade 2	Involvement of nasal plus paranasal sinuses
Grade 3	Involvement of nasal, paranasal sinuses with orbital involvement (Rhino-orbital)
Grade 4	Involvement of nasal, paranasal, sinus orbital with cerebral involvement (Rhino cerebral)

Data from the case record proforma were entered into Microsoft Excel spreadsheet version 2016 (Microsoft® Corp., Redmond, WA) and analyzed. Frequency and proportion (percentages) expressed the categorical data. The normality of continuous data was decided by plotting histograms. Non-normal and normally distributed continuous variables were expressed as median with interquartile range (IQR 25 to 75) and mean (standard deviation). For determining the statistical differences in categorical data, a Chi-square test was applied. For normally distributed continuous data, a student t-test was applied, whereas, for non-normal continuous data, the non-parametric test of Mann-Whitney U was applied. Multinomial logistic regression analysis was done to identify the predictors of mortality. P-value < 0.05 was considered significant for all statistical comparisons.

## Results

In total, 84 patients identified to have CAM were included in the analysis. All these patients were referred to us from other centers. Baseline characteristics along with details of COVID-19 are presented in Table 2. The mean age was 49.3 ± 12.1 years, and 83.3% were males. Diabetes mellitus (64.3%) and hypertension (36.9%) were the major comorbid conditions. Of the 84 patients, 86.9% had been hospitalized for COVID-19. The remaining 11 (13.09%) patients were treated under home isolation by their primary physicians before being referred for management of mucormycosis to our institute. Out of these 11 patients, 45.45% had received oral steroids, and 57.14% had diabetes. Steroid and remdesivir were administered to 83.3% and 67.9% of patients, respectively.

**Table 2:** Baseline characteristics COVID-19: coronavirus disease 2019

Parameters	Observation (n=84)
Age (years)	49.3 ± 12.1
Age groups	
≤50 years	45 (46.4)
>50 years	39 (53.6)
Gender	
Male	70 (83.3)
Female	14 (16.7)
Smoking	4 (4.8)
Comorbidities	
Diabetes mellitus	54 (64.3)
Hypertension	31 (36.9)
Ischemic heart disease	4 (4.8)
Chronic kidney disease	3 (3.6)
Hospitalized for COVID-19	73 (86.9)
Oxygen required	57 (67.9)
Laboratory investigations	
Hemoglobin (gm/dl) (n=56)	12.4 ± 2.0
Total leucocyte count (cells/cmm) (n=58)	8524.5 ± 4392.2
Platelet count (cells/cmm) (n=45)	244.9 ± 129.2
Serum ferritin (ng/ml) (n=41)	314 (140.5 to 472)
C-reactive protein (mg/L) (n=43)	12.5 (6 to 35)
D-dimer (mcg/mL) (n=51)	416 (194 to 680)
Treatment	
Remdesivir	57 (67.9)
Favipiravir	6 (14.3)
Steroid	70 (83.3)
Dexamethasone	23 (32.9)
Methylprednisolone	43 (61.4)
Deflazacort	7 (10.0)
Tocilizumab	3 (3.6)
Colchicine	1 (1.2)
Cyclosporine	1 (1.2)
Baricitinib	1 (1.2)

Details of mucormycosis in study patients are shown in Table 3. Mucormycosis was diagnosed after a median of 11 days from the onset of COVID-19. Facial pain/swelling and eye pain/swelling were the commonest complaints; 9.5% of patients had blurred vision or vision loss. Orbital and CNS involvement was seen in 29.8% and 23.8% of patients, respectively. Mean levels of HbA1c (n=56) and serum creatinine (n=51) were 8.5 ± 2.1% and 1.2 ± 1.1 mg/dl respectively. Ten (11.9%) patients developed renal dysfunction during the hospital stay. Out of these, three required dialyses. The majority of the patients were treated with liposomal amphotericin B (85.7%) along with posaconazole (65.5%). Surgical interventions were done in 95.2% of patients. Depending on the MRI grading and extension of the disease, an operative procedure was performed. For grade 1 and 2 diseases, functional endoscopic sinus surgery with paranasal sinus debridement was done with a widening of sinus drainage pathways with the removal of mucosa and involved bony parts for the disease limited to paranasal sinuses and maxilla or palate and mandible. In case of maxillary involvement, medial or total maxillectomy with ethmoidectomy with frontal exploration with sphenoidotomy, septectomy, and turbinectomy was done. For grade 3 disease (orbital involvement), surgery was extended to remove lamina papyracea with endoscopic orbital clearance with sparing of the optic nerve for patients with preserved vision or light perception. Orbital exenteration was performed when no vision or ocular movements or perception of light or complete necrosis. For grade 4 disease with intracranial extension, a neurosurgeon was involved in patient management. In patients with the spread of infection through the frontal sinus, extensive debridement with frontal bone removal by a coronal incision was done for complete removal of disease. The cavity was closed using the fat or fascia lata. In patients with infratemporal and pterygopalatine fossa involvement, extensive debridement of the skull base was done. In the presence of brain abscess, drainage and complete removal of disease and cavity closure with gel foam were done.

**Table 3:** Mucormycosis details

Parameters	Observations (n=84)
Clinical presentation	
Facial swelling/pain	18 (21.4)
Eye pain/swelling	28 (33.3)
Headache	23 (27.4)
Blurred vision / Vision loss	8 (9.5)
Nasal discharge / Blockade	12 (14.3)
Proptosis	3 (3.6)
Facial palsy	2 (2.4)
Diplopia	2 (3.5)
Central nervous system involvement	20 (23.8)
Orbital involvement	25 (29.8)
Renal dysfunction during hospitalization	10 (11.9)
Diagnosis after first onset COVID-19 symptoms (days)	11 (6.5 to 15)
Laboratory investigations	
Hemoglobin (gm/dl) (n=77)	11.8 ± 2.1
Total leucocyte count (cells/cmm) (n=77)	11192.2 ± 5360.7
Platelet count (cells/cmm) (n=72)	276.5 ± 109.1
Blood Urea (mg/dl) (n=72)	37.2 ± 31.3
Serum creatinine (mg/dl) (n=51)	1.2 ± 1.1
HbA1c (%) (n=56)	8.5 ± 2.1
MRI grade	
Grade 1 - 2	34 (40.5)
Grade 3	22 (26.2)
Grade 4	28 (33.3)
Treatment	
Intravenous	
Liposomal Amphotericin-B	72 (85.7)
Non-liposomal Amphotericin-B	4 (4.8)
Voriconazole	1 (1.2)
Oral	
Posaconazole	55 (65.5)
Isavuconazole	2 (2.4)
Voriconazole	1 (1.2)
Fluconazole	1 (1.2)
Operative procedure	80 (95.2)
Hospital stay (days)	14.9 ± 9.2

**Figure 1:**
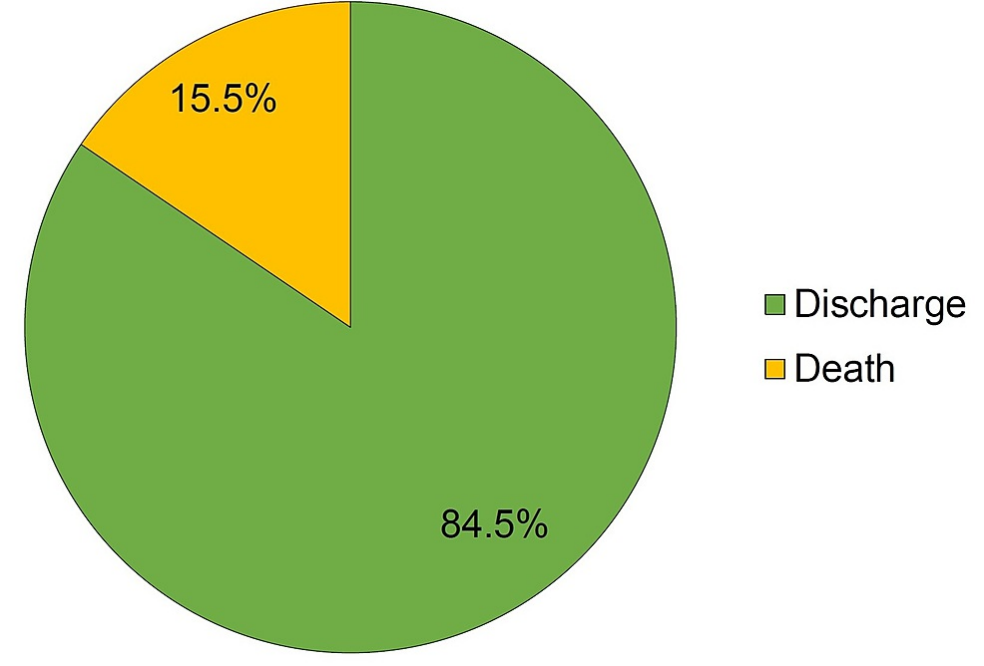
In-hospital outcome of patients with CAM

During the mean hospital stay of 14.9 ± 9.2 days, 15.5% of patients died (Figure 1). When compared to survivors, a significantly higher proportion of non-survivors had CKD (23.1% vs. 0%, p<0.0001), orbital involvement (53.8% vs. 25.4%, p=0.039), use of tocilizumab (30.8% vs. 2.8%, p<0.0001) and development of renal dysfunction during hospital stay (46.2% vs. 5.6%, p<0.0001) (Table 4). Mean levels of serum creatinine were also significantly higher in non-survivors (p<0.0001). Proportion of patients aged > 50 years (69.2% vs. 42.3%, p=0.073) and male sex (100% vs. 80.3%, p=0.079) was non-significantly higher in non-survivors than survivors. Presence of diabetes mellitus (p=0.301) and mean levels of HbA1c (p=0.329) did not differ significantly in the two groups.

**Table 4:** Factors associated with mortality n= data available for the number of patients in non-survivor group and survivor group

Parameter	Non-survivor (n=13)	Survivor (n=71)	P value
Age	53.0 ± 10.4	48.6 ± 12.3	0.230
Age >50	9 (69.2)	30 (42.3)	0.073
Male gender	13 (100.0)	57 (80.3)	0.079
Diabetes mellitus	10 (76.9)	44 (62.0)	0.301
Hypertension	3 (23.1)	28 (39.4)	0.261
Ischemic heart disease	1 (7.7)	3 (4.2)	0.589
Chronic kidney disease	3 (23.1)	0	<0.0001
O2 required	8 (61.5)	49 (69.0)	0.596
Central nervous system involvement	5 (38.5)	15 (21.1)	0.177
Orbital involvement	7 (53.8)	18 (25.4)	0.039
Renal dysfunction during hospital stay	6 (46.2)	4 (5.6)	<0.0001
Steroid use	9 (69.2)	61 (85.9)	0.138
Tocilizumab use	4 (30.8)	2 (2.8)	<0.0001
C-reactive protein (n=4 and 39)	24.5 (5 to 50.6)	12.5 (6 to 32.1)	0.702
HbA1c (n=4 and 52)	9.5 ± 4.0	8.4 ± 2.0	0.329
Hemoglobin (n=10 and 67)	12.1 ± 2.6	11.8 ± 2.0	0.715
Creatinine (n=12 and 69)	2.2 ± 1.2	1.0 ± 0.9	<0.0001
Median duration since COVID-19 diagnosis (days) (n=9 and 65)	15 (11 to 15)	10 (5 to 15)	0.096

Considering the factors identified in univariate analysis, we performed multivariate analysis (Table 5). Use of tocilizumab was associated with the highest odds of mortality in CAM patients, followed by renal dysfunction developed during the hospital stay, orbital involvement, and levels of serum creatinine.

**Table 5:** Multivariate analysis for predictors of mortality aOR: adjusted odds ratio

Parameter	aOR	95% Confidence Interval		P value
Serum creatinine	1.93	1.10	3.49	0.022
Orbital involvement	12.20	1.63	91.07	0.015
Renal dysfunction/failure during hospitalization	16.60	1.18	151.87	0.013
Tocilizumab use	25.54	1.88	347.23	0.015

## Discussion

Mucormycosis has posed a dangerous threat in India during the second wave of the COVID-19 pandemic. India contributed nearly three-fourth of the total burden of mucormycosis globally. It is probably because of the substantial presence of undiagnosed as well as uncontrolled diabetes in India [12]. Mucormycosis has variable presentations. The rhino-cerebro-orbital mucormycosis is the major form observed in this pandemic. Diagnosis is established through CT paranasal sinus and MRI brain [13]. After assessing 101 published cases of mucormycosis, Singh et al. observed that involvement of nose and sinuses (88.9%) was most common, followed by rhino-orbital (56.7%) [8]. Rhino-orbital disease can progress to CNS involvement which was observed in 28.1% of patients. Rhino-orbital-cerebral mucormycosis is a serious, severe, emergent, and fatal infection associated with high mortality. The mortality rate in our study was 15.5%. It is relatively lower compared to reported rates of 64.3% from Singh et al. [7], 47% from Pakdel et al. [9], 40% from Sarkar et al. [14], and 30.7% from Singh et al. [8]. Even in patients without COVID-19, Jiang and colleagues reported survival of only three out of 11 patients who had invasive rhino-orbital-cerebral mucormycosis [15]. It is necessary to diagnose early and initiate treatment. A delay of six days in starting the treatment increases the 30-day mortality risk by two-fold from 30% to 60% [16]. Lower mortality in our study is probably because of the implementation of protocolized management of CAM in terms of timely and effective surgical debridement (source control: 95.2%) coupled with appropriate antifungal therapy (liposomal amphotericin (>85.7%) / posaconazole (65.5%)) and diligent blood sugar monitoring with good intrahospital glycemic control with multidisciplinary medical care. 

Among various factors, CKD, orbital involvement, tocilizumab use, and renal dysfunction during the hospital stay were significantly associated with high mortality. Deutsch et al. reported that the intracranial involvement of mucormycosis increases the fatality rate to as high as 90% [17]. COVID-19 itself has a high likelihood of developing mucormycosis. The presence of hypoxia, hyperglycemia, high ferritin levels, and reduced phagocytic activity of leucocytes can contribute to the development of CAM [8]. In patients with COVID-19, CKD incidence is higher (4.09%) compared to the general population (0.46%). The presence of CKD increases mortality significantly (44.6% compared to 4.7% in those without COVID-19) [18]. This was also clear from our observation that mortality is increased in CAM patients who develop AKI. The use of tocilizumab carries the risk of infections late in the course of the disease. Pettit et al. reported infectious complications in 23% of patients after 48 hours of admission; there were three cases of invasive fungal infections in a total of 74 patients [19]. Though the use of immunomodulators is possibly indicated to be implicated in CAM, no current evidence identifies whether prior use of tocilizumab is associated with increased mortality in CAM. However, we observed significantly increased odds of in-hospital mortality when tocilizumab was used in the management of COVID-19 infection.

Among the risk factors, diabetes mellitus, especially uncontrolled diabetes, is a significant risk factor for mucormycosis [20]. Though diabetes was seen in 70.2% of patients, we found no association of diabetes with mortality. Though uncontrolled blood sugar levels are one of the risk factors for CAM, average HbA1c did not differ significantly in survivors and non-survivors. The impact of diabetes on mortality in patients with CAM needs further evaluation in a more extensive study. In addition to these factors, old age is also a factor detrimental to the outcome of CAM.

Our study was limited by retrospective design, single-center, and small sample. Though the in-hospital mortality rate was lower than most reported studies, the lower event rate makes it difficult to draw substantial conclusions for the predictors of mortality. Nonetheless, the study identified that orbital involvement and renal dysfunction to be associated with high mortality.

## Conclusions

Mucormycosis in patients with COVID-19 is a double-trouble that causes significant morbidity and morbidity. The rise in CAM in India posed a substantial threat as a higher number of cases were detrimental to the health of the individuals and community. The current study highlights that a multidisciplinary approach in CAM patients that includes timely and effective surgical debridement coupled with appropriate antifungal therapy and diligent sugar monitoring with intrahospital glycemic control may help to lower mortality. We suggest ophthalmic and brain screening early in the course of CAM patients to improve survival. In addition, comorbidities such as CKD and renal dysfunction and the use of tocilizumab can contribute to increased mortality. Control of risk factors such as diabetes, judicious use of immunomodulators to avoid immunosuppression along with early diagnosis and treatment is the key to improving survival in mucormycosis patients with COVID-19.
